# The catecholamine neurotransmitter precursor tyrosine increases anger during exposure to severe psychological stress

**DOI:** 10.1007/s00213-014-3727-7

**Published:** 2014-09-16

**Authors:** Harris R. Lieberman, Lauren A. Thompson, Christina M. Caruso, Philip J. Niro, Caroline R. Mahoney, James P. McClung, Gregory R. Caron

**Affiliations:** 1 Military Nutrition Division, US Army Research Institute of Environmental Medicine, Kansas Street, Natick, MA 01760 USA; 2 Cognitive Sciences, US Army Natick Soldier Research, Development, and Engineering Center, Kansas Street, Natick, MA 01760 USA; 3 SERE EAST, Center for Security Forces, Brunswick, ME 04011 USA

**Keywords:** Mood, Cortisol, Amino acid, Norepinephrine, Dopamine, SERE, Interrogation, Military

## Abstract

**Rationale:**

Acute stress produces behavioral and physiological changes modulated by central catecholamines (CA). Stress increases CA activity, and depletion of CA stores reduces responses to stress. Increasing CA activity by administration of the dietary amino acid CA precursor tyrosine may increase responsiveness to stress. This study determined whether tyrosine enhances the ability of humans to respond to severe stress.

**Methods:**

Severe psychological stress was generated during training at Survival, Evasion, Resistance, and Escape (SERE) School. The acute stressor consisted of two mock interrogations conducted during several days of simulated captivity. Seventy-eight healthy male and female military personnel participated in this double-blind, between-subjects study, in which they received either tyrosine (300 mg/kg, *N* = 36) or placebo (*N* = 36). Tyrosine (or placebo) was administered in food bars in two doses of 150 mg/kg each approximately 60 min before each mock interrogation. Mood (Profile of Mood States), saliva cortisol, and heart rate (HR) were assessed prior to stress exposure during a week of academic training preceding mock captivity and immediately following the mock interrogations.

**Results:**

The severe stress produced robust effects on mood (i.e., increased tension, depression, anger, fatigue, vigor, and confusion; *p* < .001), cortisol, and HR (*p* < .001). Tyrosine increased anger (*p* = .002, ANOVA treatment condition by test session interaction) during stress but had no other effects.

**Conclusion:**

Tyrosine did not alter most subjective or physiological responses to severe acute stress, but it increased ratings of anger. The modest increase in anger may be an adaptive emotional response in stressful environments.

## Introduction

Acute stress produces a range of behavioral effects in both laboratory animals and humans. In laboratory animals, acute intense physiological stressors such as cold, heat, high altitude, or psychological stressors such as restraint (Stone 1975; Gray 1982; Shurtleff et al. 1993) cause animals to become less responsive to the environment, explore less, and appear generally debilitated (Stone 1975; Gray 1982; Shurtleff et al. 1993). In humans, acute stress produces similar behavioral symptoms (Gray 1982).

Acute stress increases release and turnover of the catecholamine neurotransmitters norepinephrine (NE) and dopamine (DA) in critical areas of the brain such as the hippocampus and prefrontal cortex (Stone 1975; Anisman and Sklar 1979; Nisenbaum and Abercrombie 1993; Sudha and Pradhan 1995; Cabib and Puglisi-Allegra 1996; Goldwater et al. 2009). Release of NE as well as DA may, under certain conditions, be modulated by availability of its dietary precursor tyrosine (Wurtman and Fernstrom 1975; During et al. 1989; Lieberman 1994; Yeghiayan et al. 2001; Lieberman et al. 2005b; Fernstrom and Fernstrom 2007). Notably, stressful environments may result in a depletion of the NE precursor, tyrosine, limiting optimal synthesis of NE. This depletion can be reduced by ingestion of tyrosine, a large neutral amino acid (LNAA) found in animal and plant protein foods (Lehnert et al. 1984a; Lehnert et al. 1984b; Brady et al. 1988). Tyrosine administration can increase NE release in the brain when NE neurons are highly active, such as during acute stress (Yeghiayan et al. 2001). Thus, supplemental tyrosine can increase brain synthesis and release of catecholamines, especially norepinephrine and also perhaps dopamine, and may in this way reduce the behavioral and physiological consequences of stress (Lehnert et al. 1984a; Lehnert et al. 1984b; Banderet and Lieberman 1989; Shurtleff et al. 1993; Lieberman 1994; Deijen and Orlebeke 1994; Neri et al. 1995; Thomas et al. 1999; Magill et al. 2003; Deijen 2005; Mahoney et al. 2007). In laboratory animals, tyrosine reduces responses to stressors such as heat, tail shock, and hypobaric hypoxia (Lehnert et al. 1984a; Lehnert et al. 1984b; Lieberman 1994; Shukitt-Hale et al. 1996; Lieberman et al. 2005b).

Although standardized laboratory methods exist for inducing acute stress in human volunteers, these procedures have relatively modest effects and perhaps are not strong enough to permit detection of the effects of tyrosine in humans (Owasoyo et al. 1992). Survival, Evasion, Resistance, and Escape (SERE) School is a type of controlled military training that is highly stressful. SERE training induces biochemical, cardiovascular, and behavioral changes characteristic of acute, severe stress exposure (Morgan et al. 2000a; Morgan et al. 2002; Morgan et al. 2004; Steffian et al. 2005; Taylor et al. 2007), higher than levels typically observed in other highly stressful situations, such as skydiving (Morgan et al. 2000a; Morgan et al. 2002; Taylor et al. 2007). Therefore, using a double-blind, placebo-controlled design, we examined the effects of consuming the amino acid tyrosine, in the form of a food bar, on mood, cortisol, and heart rate in the controlled, highly stressful environment of SERE School. We hypothesized that tyrosine would reduce the behavioral and physiological consequences of acute, severe psychological stress, relative to placebo.

## Methods

A double-blind, placebo-controlled, between-subjects design was employed. This study was conducted at the US Navy SERE School in Brunswick, ME. The protocol and all procedures were approved by the National Naval Medical Center and US Army Research Institute of Environmental Medicine Institutional Review Boards and conducted in accordance with the Declaration of Helsinki. Written informed consent was obtained from all volunteers.

### Subjects

Seventy-eight healthy volunteers (72 males and 6 females; mean age 25 ± 4 years; mean body weight 80.9 ± 11.4 kg) completed the study (Table 1). Prior to admission to SERE School, all students received a medical clearance documenting their good health. All volunteers were either active duty US Navy (*N* = 59) or Marine (*N* = 19) personnel enrolled in the US Navy SERE School, Brunswick, ME. They had an average 4 years of active duty military service. Use of psychotropic medications or illegal substances is not permitted at SERE School, and random urine tests are periodically conducted on all military personnel.

**Table 1:** Volunteer demographics by treatment group

	Placebo	Tyrosine	Level of significance, *p* value
Age (years, *M* ± SD)	24.87 ± 3.36	24.85 ± 4.32	.980^a^
Height (cm, *M* ± SD)	177.72 ± 8.53	177.17 ± 7.59	.754^a^
Weight (kg, *M* ± SD)	81.57 ± 12.73	80.05 ± 10.16	.557^a^
Active duty (years, *M* ± SD)	3.97 ± 3.23	3.90 ± 3.04	.918^a^
Sex (males/females, *N*)	37/2	35/4	.237^b^

^a^ *t* test

^b^Fisher’s exact test of independence

### SERE School

Training in SERE School is required for US military personnel at high risk of capture. At the SERE School at which this study was conducted, each course lasted two weeks, and consisted of three sequential phases: (1) a week of academic classroom training, (2) several days of survival training conducted in a natural environment, and (3) the final portion of the course which was a captivity phase lasting several days. During the classroom portion, students were trained in survival, evasion, resistance, and escape techniques. During the second phase, they completed a survival field exercise. In the third phase, trainees were “captured” and applied their newly learned resistance skills in a stressful captivity environment that simulated actual captivity, including mock interrogations which generated particularly intense levels of acute stress (Morgan et al. 2000a). One objective of realistic exposure to stress is to enhance the ability to resist future stress (Morgan et al. 2000a; Morgan et al. 2002; Taylor et al. 2007).

### Procedures

Volunteers were recruited and completed a demographic questionnaire during day 1 of the course (during the academic/classroom phase of SERE School). During this phase, they practiced a standardized mood questionnaire, the Profile of Mood States (POMS), on three occasions for 5 min each, twice on day 2 and once on day 3. The final POMS practice session conducted on day 3 served as a pre-stress baseline condition (session 1). A baseline measure of heart rate was obtained on day 2 of the course. In addition, three baseline saliva samples were obtained on day 2 of the course in the morning (baseline sample “A-morning”), midday (baseline sample “B-midday”), and evening (baseline sample “C-evening”).

Subsequently, during the final phase of SERE School (the captivity phase), mood was assessed and saliva collected on three separate occasions. The first two of these test sessions (sessions 2 and 3) were conducted immediately following interrogation sessions and were separated by 5–6 h. Tyrosine or placebo was administered 1–2 h prior to both sessions 2 and 3 as described below. A fourth session (session 4) was conducted when the captivity phase was nearing completion, approximately 12 h after session 3. Additional scheduling details cannot be specified here because providing them would diminish the effectiveness of SERE training.

### Tyrosine administration

Volunteers were randomly assigned in a counterbalanced order to either tyrosine or placebo; 36 received tyrosine and 36 received placebo. Tyrosine or placebo was administered in food bars prior to both sessions 2 and 3. The bars were manufactured by the Natick Soldier Research, Development and Engineering Center (NSRDEC) and were composed of a matrix of approximately 7 % fat, 2 % protein (other than tyrosine), and 70 % carbohydrate. The total dose of tyrosine administered was 300 mg/kg; 150 mg/kg was administered in one bar given 1 h prior to the first interrogation (session 2) and a second bar was consumed 1–2 h prior to the second interrogation (session 3). The size of the food bars each volunteer received varied depending on his/her body weight. Every gram of tyrosine was administered with 5 g of the food bar matrix. For example, a 70-kg volunteer received a total of 21 g of tyrosine and 105 g of the matrix, combined for a total of 126 g. A placebo-treated volunteer of the same weight would have received 126 g of the matrix alone. This pattern and level (300 mg/kg) of dosing is similar to that used in previous tyrosine studies and substantially increases plasma tyrosine levels (Glaeser et al. 1979; Banderet et al. 1996). In one of these studies, a dose of 150 mg/kg of tyrosine increased plasma tyrosine threefold 2 h after administration and had a minimal effect on the other large neutral amino acids (LNAAs) (Glaeser et al. 1979). Banderet et al. (1996) observed even larger increases in plasma tyrosine levels when they administered tyrosine in a food bar similar to the bar used in this study. There was no discernible difference in the taste of the two treatment bars. No other food or dietary supplements were provided prior to the administration of treatment bars; water was available to SERE students throughout training.

### POMS questionnaire

The POMS is a widely used, standardized, computer- or paper-and-pencil-administered inventory of mood states (McNair et al. 1971). It provides a comprehensive assessment of a wide range of mood states and captures the fundamental domains of human affect (McNair et al. 1971). It is sensitive to a wide variety of nutritional manipulations, including tyrosine, environmental factors, sleep loss and subclinical doses of various drugs, and a variety of stressors including sleep deprivation and environmental extremes (Banderet and Lieberman 1989; Shukitt-Hale et al. 1998; Lieberman et al. 2002a; Lieberman et al. 2002b; Lieberman et al. 2002c; Scott et al. 2006; Lieberman et al. 2009; Fogt et al. 2010). It takes less than 5 min to complete. Volunteers rated a series of 65 mood-related adjectives with regard to how they were feeling “right now” on a scale of 0 (not at all) to 4 (extremely). When analyzed, the adjectives provide six independent, factor-analytically derived mood subscales (tension, depression, anger, vigor, fatigue, and confusion) (McNair et al. 1971).

### Cortisol

To assess the physiological level of stress induced by captivity, salivary cortisol was measured during academic and captivity phases of SERE School. Saliva cortisol is frequently used to assess the impact of psychological and physical stress on humans (Kahn et al. 1988; Opstad 1994; Morgan et al. 2000a; Lieberman et al. 2005a). Cortisol levels in saliva are highly correlated with those in blood (Kahn et al. 1988; Kirschbaum and Hellhammer 1989). Increased release of the hormone cortisol is the most widely accepted biological marker of activation of the hypothalamus-pituitary-adrenal axis (HPA) in humans (Laudat et al. 1988; Kirschbaum and Hellhammer 1989).

Cortisol is released in a circadian pattern, with the highest level in the morning. Therefore, during baseline testing, it was assessed three times over the course of a single day, session 1:A-morning (0600 h), session 1:B-midday (1200 h), and session 1:C-evening (1630 h), during the academic, lower-stress phase of SERE School. This permitted a comparison of cortisol release during the captivity phase to the equivalent time of day of baseline testing conducted during academic week. During the captivity phase of the study, saliva samples were collected each time mood was assessed (sessions 2, 3, and 4).

Ten milliliters of saliva was collected in cryovials during each session. Five minutes before saliva collection, volunteers rinsed their mouths with water, placed a short straw in their mouth, and allowed saliva to flow (unstimulated) into a cryovial. The samples were stored at −70 °C and shipped on dry ice to the Pennington Biomedical Research Center (Baton Rouge, LA) where they were assayed for cortisol using standard procedures.

### Heart rate

Heart rate (HR) was assessed using the Hidalgo Equivital™ Vital Sign Detection System (VSDS) (Hidalgo Ltd., Swavesey, Cambridge, UK), a chest-worn monitor designed to function in harsh environments. Baseline data were collected for at least 4 h on day 2 of the academic instructional phase of SERE School. Mean HR for this period was computed. During the captivity phase, the VSDS was placed on the volunteer’s chest before they received their first treatment bar and was removed after the second interrogation session (session 3) was completed. Peak HRs were determined for the periods during the two interrogations.

### Statistical analysis

Statistical analysis was conducted using the IBM SPSS statistical package version 19.0 (IBM SPSS, Chicago, IL). Descriptive data are presented as mean ± SEM. A two-tailed *p* value of ≤0.05 was considered statistically significant. Chi-square or Fisher’s exact test of independence were conducted to determine if treatment groups varied in demographic composition. Repeated measures ANOVA were used to determine if significant differences in mood, HR, and cortisol were present across the test sessions. A within-subjects factor of test session measured effects of SERE School over time on mood, and a between-subjects factor tested for tyrosine vs. placebo effects. Significant main or interaction effects were analyzed using the Least Significant Difference (LSD) post-hoc test.

Because cortisol is released in a manner that is dependent on time of day, separate ANOVAs for this parameter were conducted by matching and comparing equivalent times of saliva collection during academic week and captivity week. Since the initial interrogation was conducted midday for subjects in the study, the session 1:B-midday (1200 h) baseline saliva session was used for comparison to it; for the second interrogation, conducted in the evening, the appropriate comparison was the session 1:C-evening baseline saliva sample (1630 h). The final captivity week session (session 4) was conducted in the morning, so the session 1:A-morning baseline session, conducted at 0600 h, was used for comparison.

## Results

As illustrated in Table 1, there were no statistically significant differences in assignment of students to treatment condition.

### Mood

**Fig. 1:**
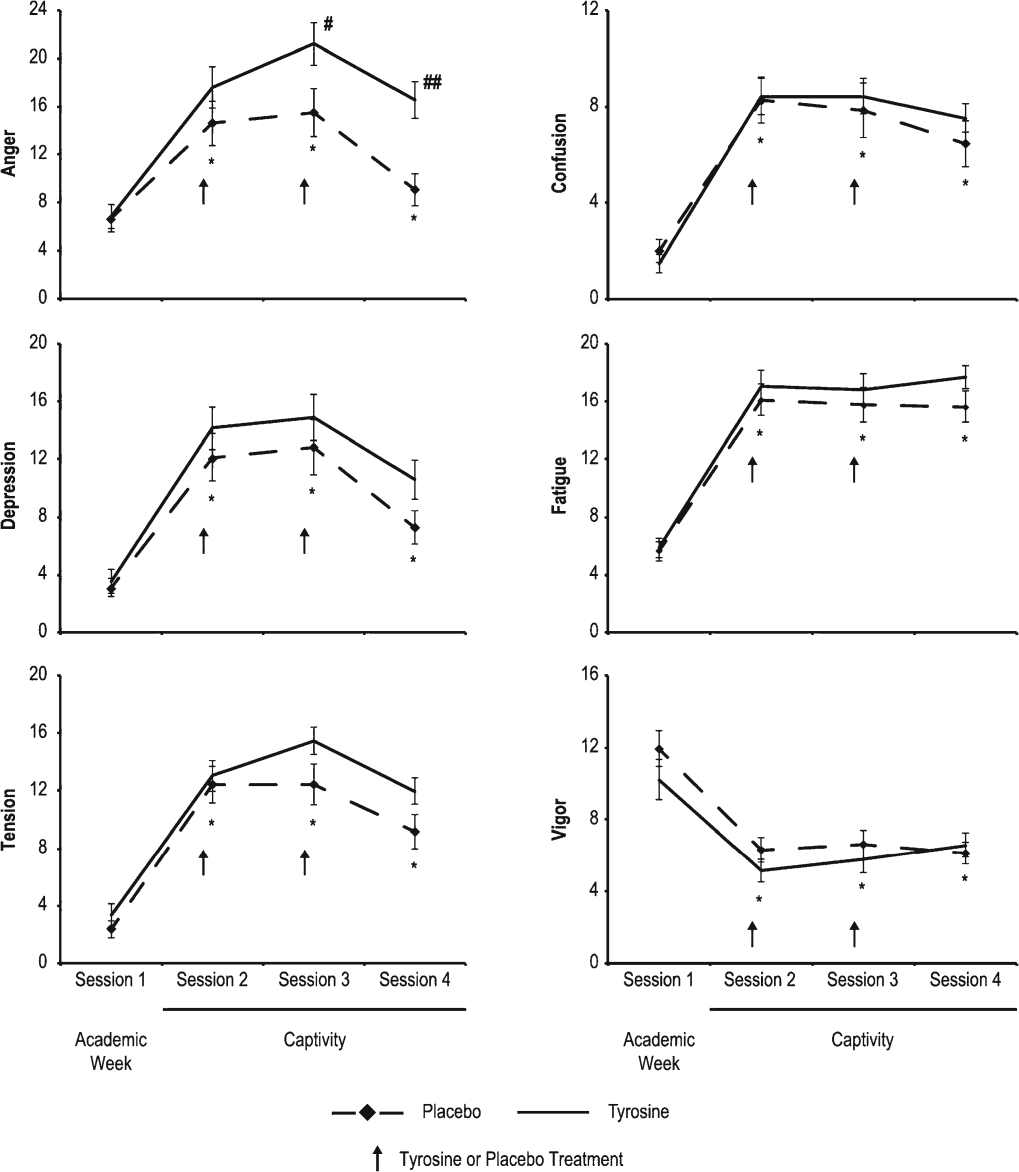
Changes in the six subscales of the POMS over the course of SERE training as a function of tyrosine vs. placebo administration. Tyrosine significantly increased the anger subscale at sessions 3 and 4 during the stressful captivity phase of SERE School. Session 1 was conducted prior to treatment administration during academic week, the low-stress portion of SERE School. Tyrosine or placebo was administered just prior to sessions 2 and 3 as shown by the *arrows*. Session 4 was conducted the following day. Significant effects of tyrosine compared to placebo on post-hoc testing at specific time points are indicated by a # (*p* < .03) or ## (*p* < .001). Significant changes on post-hoc testing when session 2, 3, or 4 was compared to session 1 (academic week-baseline) are indicated by * (*p* < .001)

Exposure to the captivity phase of SERE School significantly altered every subscale of the POMS (tension, depression, anger, fatigue, vigor, confusion, and total mood disturbance) as indicated by significant within-subject time effects on ANOVA (*p* < .001, all subscales and total mood disturbance; Fig. 1). Post-hoc testing demonstrated that baseline scores (session 1) were always significantly different from captivity week sessions, with the greatest differences at sessions 2 and 3, the sessions immediately following mock interrogations (Fig. 1). The magnitude of the changes in all mood states were generally greatest when comparing session 1 to session 3; tension increased 392 %, depression increased 317 %, anger increased 171 %, fatigue increased 183 %, vigor decreased 44 %, confusion increased 368 % (Fig. 1), and the total mood disturbance score (not shown) increased 601 %.

Tyrosine administration affected the mood state of anger as indicated by significant treatment effects between-subjects (*p* = .02) and interaction (*p* = .002) effects on ANOVA (Fig. 1). Post-hoc testing demonstrated that anger levels were greater following interrogation at session 3 (*p* = .03) and session 4 (*p* < .001) in those receiving the tyrosine-containing food bar. No other mood parameter was significantly affected by tyrosine administration (tension: between subjects [*p* = .11], interaction [*p* = .33]; depression: between subjects [*p* = .24], interaction [*p* = .48]; vigor: between subjects [*p* = .70], interaction [*p* = .06]; fatigue: between subjects [*p* = .20], interaction [*p* = .35]; confusion: between subjects [*p* = .63], interaction [*p* = .64]).

### Cortisol

**Fig. 2:**
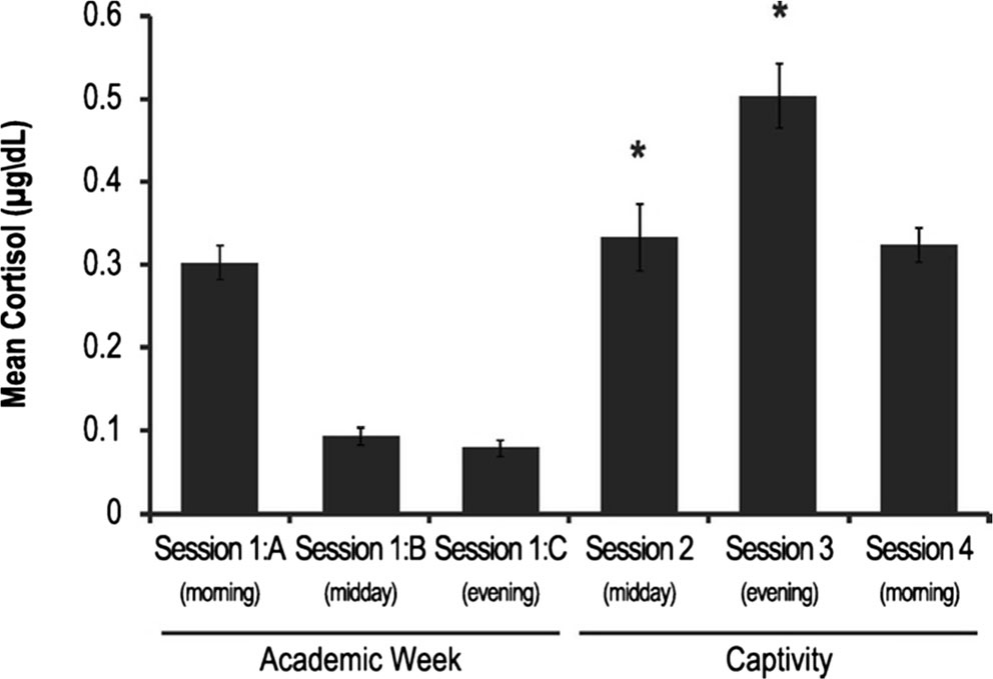
Cortisol levels (μg/dL) during academic week (session 1: A-morning, B-midday, and C-evening) and captivity (sessions 2, 3, 4). All session 1 samples (A, B, C) were obtained during baseline testing (pre-stress exposure). Sessions 2, 3, and 4 were conducted sequentially over the course of the captivity phase of SERE School. Session 2 was conducted midday immediately following the first interrogation. Session 3 was conducted immediately following the second interrogation. Session 4 was conducted in the morning when the captivity phase of SERE training was nearing completion. Due to substantial circadian variations in release of cortisol, statistical comparisons of captivity testing sessions were only conducted by comparing each session to the assessment session conducted at the equivalent time of day during academic week (*p < .001 on the within-subject factor of ANOVA)

Saliva cortisol levels were elevated after the two interrogation sessions compared to the appropriate time-matched baseline measures (*p* < .001, Fig. 2). The final captivity week session (session 4) was not significantly different from the appropriate baseline control measure (Fig. 2). There were no significant differences on ANOVA when cortisol levels of volunteers receiving tyrosine-containing food bars were compared to those of volunteers receiving placebo food bars.

### Heart rate

**Fig. 3:**
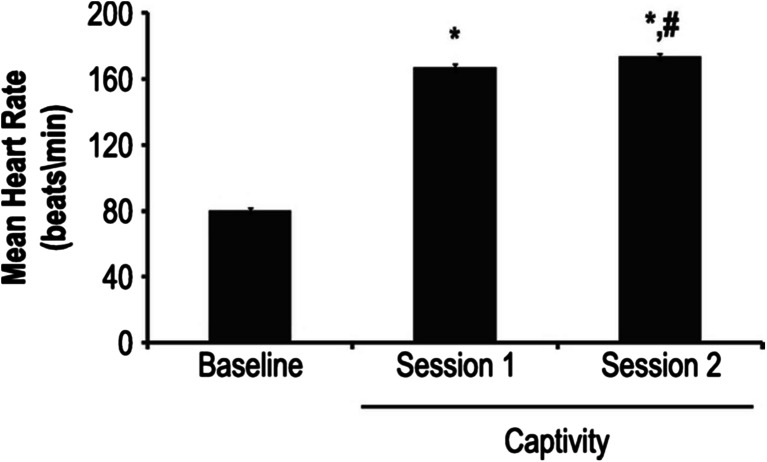
Mean HR (beats/min) during baseline testing conducted during the academic non-stressful portion of SERE School and peak heart rates during sessions 2 and 3 when interrogations were being conducted. On post-hoc testing, mean peak session 2 and session 3 HRs were significantly higher than baseline (*p < .001). Session 3 HR was significantly higher than Session 2 HR (#*p* = .008)

Mean baseline HR was 80.1 ± 1.6 beats/min. Mean peak HR during the first interrogation session was 166.7 ± 2.4 beats/min and during the second interrogation session, 173.5 ± 1.9 beats/min (Fig. 3). There was a significant effect of test session on HR (*p* < .001). Post-hoc testing demonstrated that HR was lower during baseline testing (*p* < .001) compared to either interrogation session, and during interrogation session 2, HR was higher than interrogation session 1 (*p* < .008, Fig. 3). There were no significant differences in HR between tyrosine- and placebo-treated volunteers on ANOVA.

## Discussion

This study demonstrates that during severe psychological stress, double-blind administration of the dietary catecholamine neurotransmitter precursor tyrosine in a food bar increases the mood of anger as measured by a validated, self-report mood questionnaire. Tyrosine’s effects on anger were robust and consistent across multiple test sessions. At the baseline test session, the tyrosine and placebo groups displayed equivalent and low levels of anger. Immediately after mock interrogations, considered the most stressful experiences at SERE School (Morgan et al. 2000a), the mood of anger substantially increased in both groups, but the increase was significantly greater in the tyrosine-treated group. The mood of anger may, under certain circumstances, including SERE School, be a justified and appropriate response to provocation and has been found to be associated with the stress of SERE School (Taylor et al. 2009). It is reported that short-term anger provides a sense of control and optimism in healthy adults who are exposed to a stressful situation (Lerner et al. 2007). Anger may enhance SERE students’ ability to effectively respond to the stress of mock captivity, including mock interrogations, consistent with other positive effects of appropriately focused anger (Lerner and Keltner 2001). However, an increase in anger may not be adaptive and, in certain circumstances, may be counterproductive.

Future studies should investigate whether increased anger is beneficial in SERE School and other circumstances or has negative consequences, and whether other mood states are altered by tyrosine administration. Information on other outcomes related to coping with stress, such as ability to learn coping strategies taught at SERE School and speed of recovery, would also help establish whether tyrosine is beneficial. We are not aware of any evidence that tyrosine or any other food component or drug directly affects the mood state of anger, but tyrosine has never been tested in an environment that induces high levels of anger as well as intense levels of psychological stress.

### Comparison to other stress paradigms

Concomitant assessment of the stress hormone cortisol, heart rate, and self-reported tension-anxiety (POMS) confirmed that very high levels of stress were induced by the SERE environment, especially during mock interrogations, and illustrate SERE School’s uniqueness and robustness. The changes in these parameters we observed exceeded changes typically observed in other acutely stressful situations such as skydiving, when patients undergo major surgery, when physicians perform difficult surgical procedures, or in laboratory simulations of psychological stress (Kirschbaum et al. 1993; Arora et al. 2010a; Arora et al. 2010b). For example, peak HR during the second mock interrogation reached a mean peak level of 173.5 ± 1.9 beats/min. This is an extraordinarily high level for individuals not engaged in an intense aerobic activity. We are not aware of any studies of psychological stress where peak HR levels of this magnitude have been observed. Furthermore, in our study, cortisol was elevated by a factor of 5 during the second interrogation (session 3) relative to the comparable academic week session (the session 1:C-evening measurement). In a study using the Trier Social Stress Test, a widely used stress paradigm, saliva cortisol was elevated by a factor of 2 or 3 (Kirschbaum et al. 1993).

The nature and magnitude of the level of stress we observed are consistent with previous studies of SERE School which observe very high levels of cortisol and changes in other stress hormones and cardiovascular function (Morgan et al. 2000a; Morgan et al. 2000b; Morgan et al. 2001; Morgan et al. 2002; Morgan et al. 2004; Taylor et al. 2007). The high level of stress induced by the captivity phase of SERE School indicates that it is comparable to the stress of actual combat (Morgan et al. 2000a; Morgan et al. 2000b; Morgan et al. 2001; Morgan et al. 2002; Morgan et al. 2004; Taylor et al. 2007). Future research should utilize the unique opportunity provided by SERE training to examine various interventions that could prevent the long-term adverse consequences of exposure to severe stress such as post-traumatic stress disorder (PTSD) and depression. In addition, the large number of military personnel undergoing such training provides an opportunity for studying how individual differences, such as personality and genetic variation, influence the stress response.

### Central catecholaminergic systems, learned helplessness and anger

Central noradrenergic systems are critical in the response to acute stress (Stone 1975; Nisenbaum and Abercrombie 1993; Sudha and Pradhan 1995; Dronjak et al. 2004; Morilak et al. 2005; Goldwater et al. 2009) and sensitive to treatment with exogenous tyrosine (Lehnert et al. 1984a; Brady et al. 1988; Lieberman 1994). Dopamine also increases during acute stress and may be precursor loading-sensitive, but the evidence is limited and inconsistent (Stone 1975; During et al. 1989; Westerink and De Vries 1991). During acute stress, tyrosine administration increases central noradrenergic activity and the ability of humans and other animals to appropriately respond to various stressors (Lieberman 1994). Inability to appropriately respond to acute, uncontrollable stress has been termed learned helplessness and is associated with reduced central noradrenergic activity (Gray and McNaughton 1996). Learned helplessness is one potential outcome of captivity (Steffian et al. 2005). In a standard model of depression, the forced swim test, animals treated with tyrosine, compared to placebo, swim for longer periods of time, an indication of enhanced resistance to acute stress (Rauch and Lieberman 1990; Yeghiayan et al. 2001). For humans in the SERE environment, tyrosine may have parallel effects, potentially increasing the ability to resist by increasing anger. Anger may be an appropriate response to the stress of the forced captivity and interrogation at SERE School, and we hypothesize that tyrosine, dietary precursor of NE, increases the magnitude of this response by increasing central NE levels, but note that changes in DA or both DA and NE may be responsible for the changes we observed.

The regulation of anger is a high-level cortical process, and the primary source of noradrenergic input to cerebral cortex is the locus coeruleus (LC) (Aston-Jones and Cohen 2005). The LC-NE system, which has widely distributed projections to the cortex, is associated with a range of critical behavioral functions including arousal, emotion, attention to task-focused behaviors, and motivation (Aston-Jones and Cohen 2005). Therefore, the changes in anger we observed due to tyrosine administration suggest that anger is regulated in part by NA neurons of the LC. This hypothesis is consistent with literature indicating that the LC innervates the orbitofrontal cortex (OFC), the brain region most closely associated with the modulation of anger (Morecraft et al. 1992; Murphy et al. 2003; Pichon et al. 2008).

### Study weaknesses

One weakness of this study, which is typically associated with realistic field studies, is uncontrolled variation in the testing environment and inability to include other dependent measures such as blood sampling or a measure of central or peripheral catecholamine levels. A measure of central catecholaminergic activity, such as collection of cerebrospinal fluid which contains central CA, would be particularly revealing (Watson and Wilk 1975).

## Conclusion

In conclusion, this double-blind, placebo-controlled study demonstrates that the administration of the amino acid tyrosine in the form of a food bar increases the mood of anger, a potentially appropriate response to the severe psychological stress of SERE School. Tyrosine may be an effective intervention when individuals are exposed to acute, severe psychological stress, but additional research is required to replicate and extend these findings. In addition, this study confirms and extends previous research on the nature and magnitude of the psychological stress induced by SERE School. Changes in HR may be the best overall indicator of peak levels of stress during SERE School since it can be assessed with unobtrusive ambulatory monitors during actual interrogations. This study also provides new information on simultaneously acquired behavioral, physiological, and biochemical measures during acute severe psychological stress.
